# SOCS3 Expression Correlates with Severity of Inflammation, Expression of Proinflammatory Cytokines, and Activation of STAT3 and p38 MAPK in LPS-Induced Inflammation *In Vivo*

**DOI:** 10.1155/2013/650812

**Published:** 2013-09-02

**Authors:** João Antônio Chaves de Souza, Andressa Vilas Boas Nogueira, Pedro Paulo Chaves de Souza, Yeon Jung Kim, Caroline Silva Lobo, Guilherme José Pimentel Lopes de Oliveira, Joni Augusto Cirelli, Gustavo Pompermaier Garlet, Carlos Rossa

**Affiliations:** ^1^Department of Diagnosis and Surgery, School of Dentistry at Araraquara, Universidade Estadual Paulista (UNESP), Rua Humaitá, 1680-Centro, 14801-903 Araraquara, SP, Brazil; ^2^Department of Physiology and Pathology, School of Dentistry at Araraquara, Universidade Estadual Paulista (UNESP), 14801-903 Araraquara, SP, Brazil; ^3^Department of Implantology, University of Santo Amaro, 04743-030 Santo Amaro, SP, Brazil; ^4^Department of Biological Sciences, School of Dentistry at Bauru, University of São Paulo (USP), 17012-901 Bauru, SP, Brazil

## Abstract

SOCS3 is an inducible endogenous negative regulator of JAK/STAT pathway, which is relevant in inflammatory conditions. We used a model of LPS-induced periodontal disease in rats to correlate SOCS3 expression with the inflammatory status. *In vitro* we used a murine macrophage cell line to assess the physical interaction between SOCS3 and STAT3 by coimmunoprecipitation. 30 ug of LPS from *Escherichia coli* were injected in the gingival tissues on the palatal aspect of first molars of the animals 3x/week for up to 4 weeks. Control animals were injected with the vehicle (PBS). The rats were sacrificed at 7, 15, and 30 days. Inflammation and gene expression were assessed by stereometric analysis, immunohistochemistry, RT-qPCR, and western blot. LPS injections increased inflammation, paralleled by an upregulation of SOCS3, of the proinflammatory cytokines IL-1**β**, IL-6, and TNF-**α** and increased phosphorylation of STAT3 and p38 MAPK. SOCS3 expression accompanied the severity of inflammation and the expression of proinflammatory cytokines, as well as the activation status of STAT3 and p38 MAPK. LPS stimulation in a macrophage cell line *in vitro* induced transient STAT3 activation, which was inversely correlated with a dynamic physical interaction with SOCS3, suggesting that this may be a mechanism for SOCS3 regulatory function.

## 1. Introduction

Periodontal diseases are chronic inflammatory conditions that represent the most prevalent bone lytic disease in humans and, in its wide spectrum of severity, affect most of the human population. Its initiation and progression occur as a consequence of the host immune-inflammatory responses to bacteria in the dental biofilm. These responses are initiated by the recognition of microbial-associated molecular patterns by innate immune receptors, such as toll-like receptors (TLRs) and nucleotide oligomerization domain proteins (NOD). Lipopolysaccharide (LPS) is considered one of the main virulence factors of Gram-negative bacteria associated with periodontal diseases, and it is recognized mainly by TLR4 and TLR2. Upon LPS-binding, immune and resident cells of the periodontal microenvironment produce increased levels of various proinflammatory cytokines [1, 2]. Although inflammation is an essential component of the host response to microbial challenge, excessive cytokine production results in degradation of the soft and hard tissues of the periodontium, which are the hallmarks of destructive periodontal disease [3].

The pathway of Janus kinase (JAK) and signal transducer and activators of transcription (STAT) is essential for the signaling of cytokines and other stimuli that regulates inflammatory gene expression and may represent a key mechanism by which cytokines contribute to the progression of inflammatory diseases. The binding of a ligand to type 1 or type 2 cytokine receptors activates the associated JAK, which phosphorylates the cytoplasmic domain of the receptor to allow the recruitment and tyrosine phosphorylation of STATs. Activated STATs dimerize and translocate to the nucleus, where they act as transcription factors to regulate gene expression by binding to specific DNA motifs on the promoter region of the various genes [4–6].

Strict mechanisms of cytokine-signaling control are essential for ensuring an appropriate response through JAK/STAT pathway. Members of the suppressors of cytokine signaling (SOCS) family, which comprise eight proteins, are inducible endogenous regulators of the JAK/STAT pathway. These SOCS proteins can be induced in response to a wide range of cytokines with pro- and anti-inflammatory activities [7, 8]. Among the SOCS family members, SOCS1 and SOCS3 are the best characterized in terms of their abilities to regulate proinflammatory cytokine signaling. SOCS1 and SOCS3 are negative feedback regulators of STAT1 and STAT3, respectively, and can inhibit JAK activity by different mechanisms: SOCS1 binds to JAKs, through the Src homology 2 (SH2) domain and proximal kinase inhibitory region, whereas SOCS3 is recruited by phosphotyrosine residues of the intracellular domain of the cytokine receptor and also inhibits JAK activity [9, 10]. It has been suggested that SOCS proteins can also inhibit the activity of STATs by direct physical interaction [10]. 

The signaling mechanisms controlling the cytokine network in periodontal disease are still poorly understood; however, it has been shown that SOCS proteins are expressed in established periodontal lesions and may play a role in the outcome of inflammatory reaction [11]. In this study we determined the kinetic of SOCS3 expression in a LPS model of experimental periodontal disease and correlated its expression pattern with dynamics of the inflammatory reaction, as assessed histologically/stereometrically and by the expression of pro- and anti-inflammatory cytokines. To obtain insight into the mechanism of SOCS3-mediated regulation of STAT3, we performed *in vitro* experiments in a murine macrophage cell line. 

## 2. Materials and Methods

### 2.1. *In Vivo* Experimental Periodontal Disease Model

Male Wistar rats weighing approximately 250 g were divided into two experimental groups: E—Experimental group (*n* = 27) received 3 injections/week of 3 *μ*L of a 10 mg/mL (30 *μ*g/injection) suspension of bacterial LPS on the palatal aspect of the upper molars. C—Control group (*n* = 9)—received 3 injections/week of 3 *μ*L of the vehicle (PBS) used to resuspend the LPS on the palatal aspect of upper molars. The animals were anesthesized with isoflurane, placed on a surgical table for the injections.

After 7, 15, and 30 days of the start of the injections, 3 animals from the control group and 9 animals from the experimental group were sacrificed in each period by anesthetic overdose. The maxillary jaws were hemisected, and half of the block samples including molars with their surrounding tissues were submitted to routine histological processing for stereometry and immunohistochemistry. The other half of the blocks had the gingival tissue on the palatal aspect of the first molars carefully dissected for extraction of total RNA and protein for RT-qPCR and western blot, respectively. 

This study was carried out in accordance with the principles stated by the Brazilian College of Animal Experimentation and was approved by the Ethical Committee on Animal Experimentation (protocol number 23/2007) of the School of Dentistry at Araraquara, UNESP.

### 2.2. *In Vitro* Experiment

Raw 264.7 macrophages were grown in alpha-MEM containing 100 IU/mL penicillin, 100 ug/mL streptomycin, 2 mM of L-glutamin, and 10% heat-inactivated fetal bovine serum (FBS) (Gibco-Invitrogen Corp). 2 × 10^6^ cells were plated on 100 mm dishes, allowed to attach for 24 h, washed with PBS three times, and deinduced in culture medium containing 0.3% FBS for 4 h. These cells were stimulated with 10 *μ*g/mL of *Escherichia coli* LPS (Sigma Chem Co). Negative controls were treated with the corresponding volume of the vehicle (PBS). Cell lysates were harvested after 10 and 60 min by scraping the cell monolayer in 500 *μ*L of proprietary lysis buffer, according to the instructions provided by the supplier of the coimmunoprecipitation kit (Pierce Biotechnology, Thermo-Fisher Scientific). These samples were stored at −80°C until use.

### 2.3. Stereometric Analysis

Tissue blocks were fixed in 4% buffered formalin for 48 h, decalcified in EDTA (10%, 0.5 M) for 3 months at room temperature, and embedded in paraffin. Serial sections of 5 *μ*m were obtained in the buccal-palatal direction and stained with hematoxylin and eosin. The images were taken using a light microscope (LEICA microsystem GmbH, Wetzlar, Germany). A 32400 *μ*m^2^ grid with 9 × 4 squares of 30 *μ*m (in scale with the 40x magnification used in all images) was constructed using an image editing software (Adobe Photoshop CS5) and overlaid on the digital images obtained from the histological sections. The region of interest for the analysis was represented by the whole grid, which was positioned in a submarginal area of the palatal surface, representing the connective tissue subjacent to the gingival sulcus (the apical border of the junctional epithelium and tooth structure were used as upper and lateral limits of the grid, resp.). A single examiner, who was previously trained and calibrated and blind to the purpose of the experiment, performed the stereometric analysis using a point-counting technique. The following structures observed on each intersection point of the grid were recorded: fibroblastic (elongated morphology) cells, extracellular matrix, vascular structures, and inflammatory (rounded morphology) cells. The presence of each structure was expressed as a percentage of the total area analyzed in accordance with Odze et al. [12].

### 2.4. Immunohistochemistry Analysis

Semiserial buccal-palatal sections (5 *μ*m thick, spaced 20 *μ*m between sections) were mounted on silanized slides (Dako A/S, Denmark) and immunohistochemical staining for SOCS3 was performed using anti-rat SOCS3 antibodies (Santa Cruz Biotechnology #sc-9023) raised in rabbit (1 : 300 dilution). Negative control sections were incubated with PBS (omission of primary antibody) to assess background staining. Biotinylated immunoglobulin (ABC kit Dako A/S, Denmark) was used as secondary antibody followed by incubation with avidin-biotin peroxidase complex (ABC kit Dako, Glostrup, Denmark). Diaminobenzidine (DAB, Dako A/S, Denmark) was used as a chromogen substrate. All sections were counterstained with Carrazi's hematoxylin and mounted with permount. Photomicrographs were taken using a light microscope (LEICA microsystem GmbH, Wetzlar, Germany). The immunohistochemical analysis was performed by H-score method [13] by an experienced pathologist, who was blind to primary antibody and experimental groups.

### 2.5. Quantitative Reverse-Transcription Real-Time PCR

Total RNA was extracted from tissue samples using an affinity column system (RNAqueous-4PCR, Ambion Inc.) according to the manufacturer's protocol. The quantity and purity of total RNA were determined by UV spectrophotometry and by the 260/280 nm ratio, respectively. RNA integrity of a subsample was confirmed by electrophoresis in formaldehyde agarose gels. Four hundred ng of total RNA was converted into cDNA with random hexamer primers and moloney leukemia virus reverse transcriptase in a reaction volume of 20 *μ*L (High capacity cDNA synthesis kit, Applied Biosystems).

The qPCR reactions were performed in a 20 *μ*L volume reaction including TaqMan qPCR master mix (Applied Biosystems), diluted cDNA, deionized water, and rat-specific predesigned and optimized pairs of primers and probe (TaqMan gene expression assays) (Table 1). The determination of the relative levels of gene expression was performed using the cycle threshold method and normalized to the housekeeping gene GAPDH, which was not altered by the experimental conditions. Results are represented as the mean mRNA expression from duplicate measurements of 6 to 9 samples from different animals in each period, normalized by the internal control (GAPDH) and expressed as fold change over the levels of expression of the normalized target gene determined in cDNA samples prepared from healthy control gingival tissues.

### 2.6. Western Blot and Coimmunoprecipitation

Expression of SOCS3 and activation of STAT3 were assessed using samples of total protein extracted from gingival tissues collected from rats sacrificed in the different experimental periods. Proteins were extracted using a detergent-based extraction buffer (T-PER, Tissue Protein Extraction Reagent—Pierce) in the presence of protease and phosphatase inhibitors, and concentrations were measured using the Bradford technique (Bio-Rad Laboratories). Thirty *μ*g of the proteins was diluted in SDS-containing sample buffer and 0.1 M DTT, heat-denatured, and loaded on 10% Tris-HCl polyacrylamide gel for electrophoretic separation. The proteins were then blotted onto a 0.2 *μ*m nitrocellulose membrane and subsequently blocked with 5% nonfat milk in Tris-buffered saline (TBS). For detection, the membranes were incubated overnight with the primary polyclonal antibodies (Santa Cruz Biotechnology, except p38—Cell Signaling) overnight-diluted 1 : 100 (p-STAT3 no. sc-7993, Santa Cruz Biotechnology, and p-p38 no. 9211, Cell Signaling), 1 : 200 (SOCS3 no. sc-9023 and STAT3 no. sc-7179, Santa Cruz Biotechnology), or 1 : 500 (GAPDH no. sc-166545, Santa Cruz Biotechnology) at 4°C. After removal of primary antibodies and 45 min washing in TBS, the membranes were incubated with the appropriate species-specific secondary antibodies conjugated with HRP diluted 1 : 2000 in 5% nonfat milk in TBS. Detection of target proteins GAPDH, SOCS3, phosphorylated p38, and total and phosphorylated STAT3) bands was carried out on radiographic film by using a chemiluminescence system (Pierce ECL Western Blotting Substrate). The coimmunoprecipitation assay was performed using cell lysates of LPS- and vehicle-stimulated raw 264.7 cells, according to the instructions of the supplier of the kit (Pierce Co-Immunoprecipitation kit, Thermo Scientific). Briefly, this kit uses an amine-modified affinity column system that allows the detection of native protein complexes and prevents the elution of the primary antibody (SOCS3 polyclonal rabbit IgG, cat no. sc-9023, Santa Cruz Biotechnology) with the immunoprecipitated proteins by covalent bonding. Initially, free amines and carrier proteins were removed from the primary antibody suspension by another affinity column system (Pierce Antibody Clean-Up kit, Thermo Scientific), and then 10 ug of the primary antibody was immobilized on the affinity columns. Negative controls were columns with 10 ug of rabbit irrelevant IgG and “empty” columns, without any bound antibody. A total of 500 ug of cell lysate was loaded onto the columns and incubated under constant agitation for 12 h at 4°C. After performing the washing steps following the instructions of the supplier, the proteins were eluted and prepared for SDS-PAGE as described previously and the membranes probed for total STAT3 (#sc-7179, Santa Cruz Biotechnology).

### 2.7. Statistical Analysis

For qPCR and stereometry, statistical analysis of multiple experimental periods and groups was performed using one-way analysis of variance followed by Tukey test. Results are expressed as means ± standard error of means (SEM). For immunohistochemistry (H-score) statistical analysis was performed using Kruskall-Wallis followed by Dunn test. The associations between SOCS3 expression at mRNA and protein level and inflammation and cytokine gene expression were assessed by determining the Pearson *r* correlation coefficients. *P* < 0.05 was considered statistically significant.

## 3. Results

### 3.1. LPS-Induced Inflammation Is Sustained over the 30-Day Experimental Period

**Figure 1:**
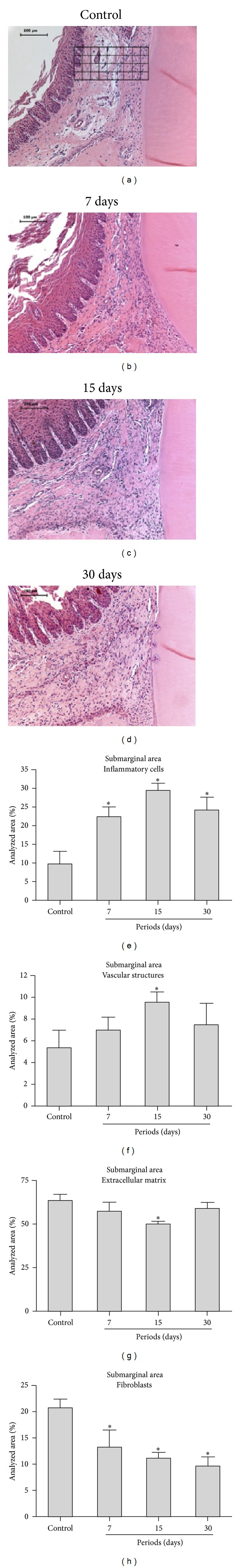
Representative images and stereometric analysis of each experimental period (7, 15, and 30 days) for the LPS-injected tissues (experimental group). A single representative image of the control group is shown, as no important changes were noted in this group in the different experimental periods (data not shown). The general characteristics of the inflammatory reaction include increase in inflammatory-like cell density and vascular structures, and a decrease in the number of fibroblast-like cells and extracellular matrix content was observed in all experimental periods. The image of a control site depicts the placement of the 3240 *μ*m^2^ grids on the submarginal area limited coronally by the apical border of the junctional epithelium and laterally by the tooth structure. Stereometric analysis indicates that LPS injections caused a sustained inflammatory reaction. The relative presence of inflammatory cells, vascular structures, fibroblasts, and extracellular matrix in 32400 *μ*m^2^ area (schematically shown on (a)) was analyzed. The severity of inflammation was significantly higher in comparison to the control group throughout the 30-day experimental period. *Significant differences compared with control groups (*P* < 0.05, ANOVA, and post hoc Tukey for pairwise comparisons).

LPS from *E. coli* (30 *μ*g per injection) caused a significant increase in the number of inflammatory cells and vascular structures already at 7 days after the first injection (Figures 1(a) and 1(b)). These inflammatory changes were sustained throughout the 30-day experimental period (Figures 1(c) and 1(d)). At 30 days, osteoclasts resorbing the bone crest could be observed, the hallmark of destructive periodontal disease (Figure 1(d)).

The stereometric analysis confirmed the increased number of inflammatory cells (defined as small, round with intensely stained nuclei) starting at 7 days (after 3 LPS injections) that was sustained at the 15- and 30-day periods (Figure 1(e)). The area covered by vascular structures also increased, and the difference was statistically significant in comparison with the control group at 15 days (Figure 1(f)). In contrast, the area covered by extracellular matrix was significantly decreased at 15 days in the experimental group (Figure 1(g)). This decrease on extracellular matrix was accompanied by a marked reduction on the proportion of fibroblastic cells (defined as spindle-shaped, elongated cells with less intense staining in the nuclei), which was also observed throughout the 30-day experimental period (Figure 1(h)).

### 3.2. Gene Expression of SOCS3 Paralleled the Increase in the Expression of Proinflammatory Cytokines in the LPS Model

**Figure 2:**
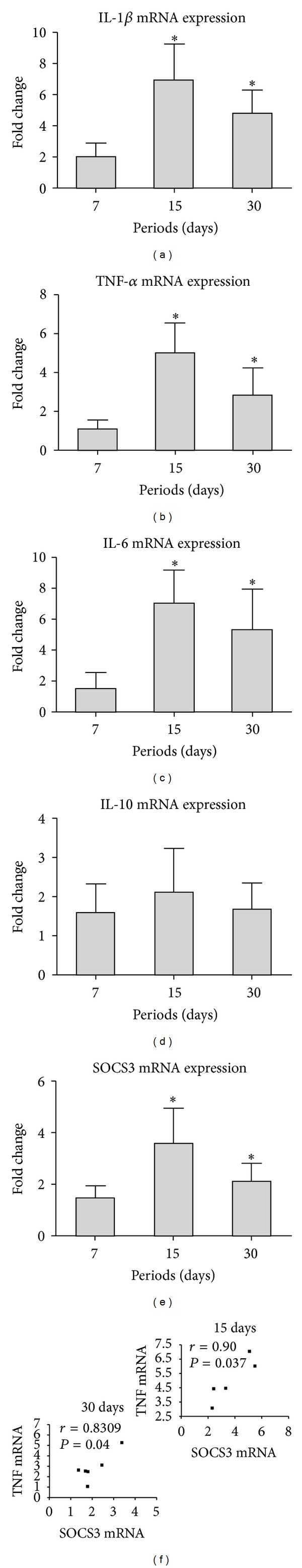
Profile of the gene expression of SOCS3, proinflammatory cytokines IL-1*β*, TNF-*α*, and IL-6 and anti-inflammatory cytokine IL-10 during the course of LPS-induced periodontal disease. Expression of all proinflammatory cytokines and SOCS3 was increased at the 15- and 30-day experimental periods, with a peak in this increase at 15 days. No regulation of the anti-inflammatory cytokine IL-10 was observed in any of the experimental periods. mRNA expression was normalized to the expression of the housekeeping gene GAPDH. The bars represent mean fold changes, and the vertical lines represent the standard deviation of the mean fold change of six animals in each experimental group in comparison to untreated control. *Significant differences (*P* < 0.05, ANOVA, and post hoc Tukey tests for pairwise comparisons).

Gene expression of candidate inflammatory cytokine genes in the gingival tissues was determined at 7, 15, and 30 days. mRNA expression levels of IL-1b, TNF-*α*, and IL-6 in LPS-injected tissues were significantly increased at 15 and 30 days in comparison with the control group (Figures 2(a) to 2(c)), with peak expression of these genes at the 15-day experimental period. Gene expression of anti-inflammatory IL-10 was not regulated in this model (Figure 2(d)). Expression levels of SOCS3 gene paralleled the expression of inflammatory cytokines and also peaked at 15 days (Figure 2(e)). There was significant correlation (*P* < 0.05) between SOCS3 mRNA and TNF-*α* mRNA at 15 and 30 days (Figure 2(f)). In spite of a decrease at 30 days, gene expression of SOCS3 remained significantly higher in the LPS-injected tissues (Figure 2(e)). 

### 3.3. Increased Activation of STAT3 and p38 MAPK in the LPS Model of Periodontal Disease Is Also Positively Correlated with SOCS3 Protein Expression

**Figure 3:**
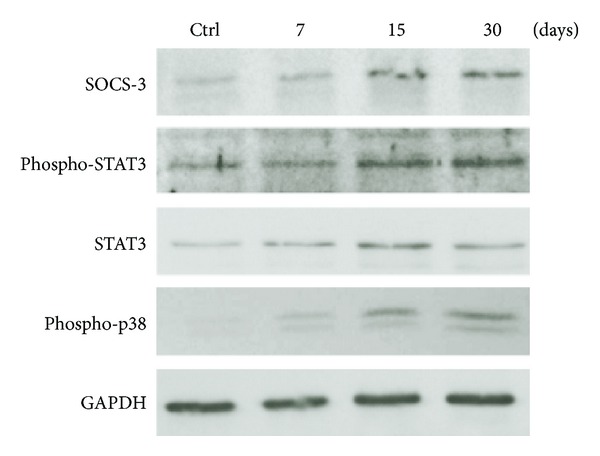
Western blot analysis of SOCS3, STAT3, and p38 MAPK protein expression in the LPS model of periodontal disease. Total protein was extracted from gingival tissue samples obtained from LPS and control sites at 7-, 15-, and 30-day periods. Activation of STAT3 and p38 as well as the expression of SOCS3 was determined. Increases in expression of SOCS3 and in the activation of STAT3 and p38 MAPK were observed at 7-, 15-, and 30-day experimental periods. Note that total STAT3 (including phosphorylated and nonphosphorylated forms) was also increased in the experimental group. The images are representative of the results obtained using samples from three different animals per period.

**Figure 4:**
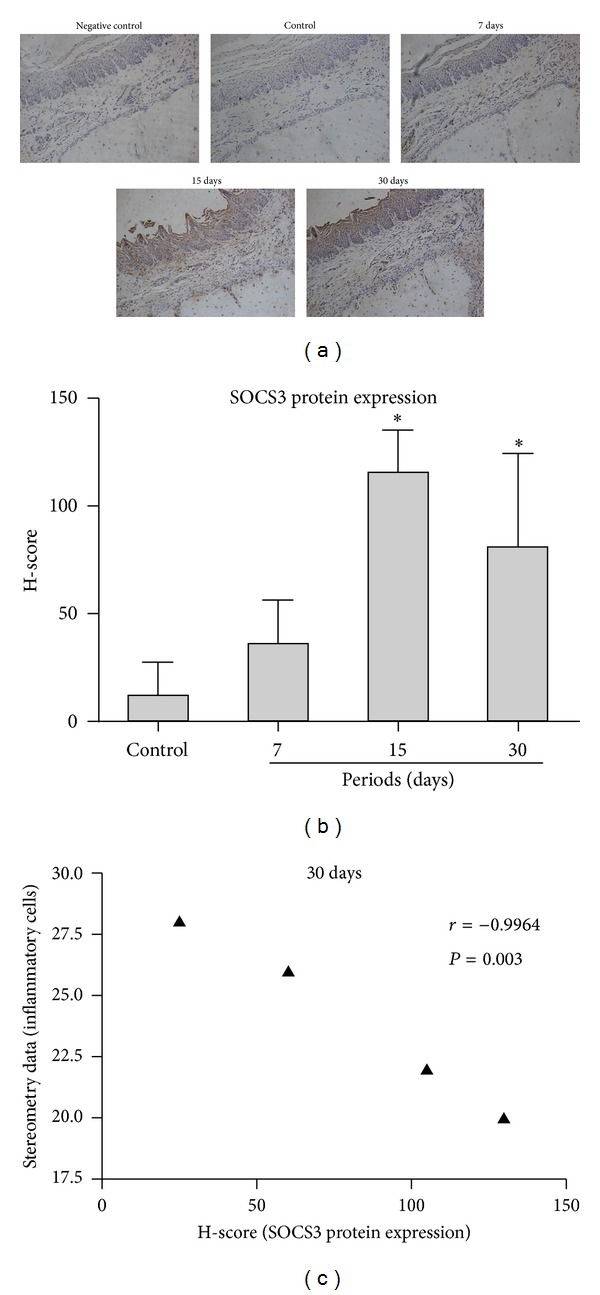
Immunohistochemical analysis revealed an increased number of SOCS3-positive cells after 7, 15, and 30 days after LPS injection. This increase reached statistical significance in comparison with gingival tissues of the control group at 15 and 30 days, as indicated by H-score analysis. Note that SOCS3-positive cells were located primarily near blood vessels and on the surface of alveolar bone. *Significant differences compared with control groups (*P* < 0.05).

LPS injections activated STAT3 and p38 MAPK signaling in the gingival tissues in all experimental periods (Figure 3). Interestingly, the increase in the activation of STAT3 was accompanied by an increase in the total protein levels of these transcription factors, as demonstrated by the western blot using a specific antibody against total STAT3. The expression of SOCS3 protein also increased at 7, 15, and 30 days after the start of LPS injections (Figure 3). In agreement with the assessment of SOCS3 protein in gingival tissue lysates, immunohistochemical analysis revealed an increased number of SOCS3-positive cells 7, 15, and 30 days after LPS injection (Figure 4(a)), which was significantly greater in comparison with the control, PBS-injected tissues at 15- and 30-day periods, as indicated by H-score analysis (Figure 4(b)). Interestingly, most SOCS3-positive cells were located near blood vessels in the connective tissue in the proximity of alveolar bone, suggesting that the LPS and/or the endogenously-produced inflammatory mediators induced SOCS3 expression in inflammatory cells and osteoblasts. Interestingly, there was a significant (*P* < 0.01) negative correlation between SOCS3 protein expression and inflammation assessed by stereometry, supporting the role of SOCS3 as an endogenous negative regulator in an inflammation-induced feedback loop (Figure 4(c)).

### 3.4. SOCS3 Physically Interacts with STAT3 in LPS-Stimulated Macrophages

**Figure 5:**
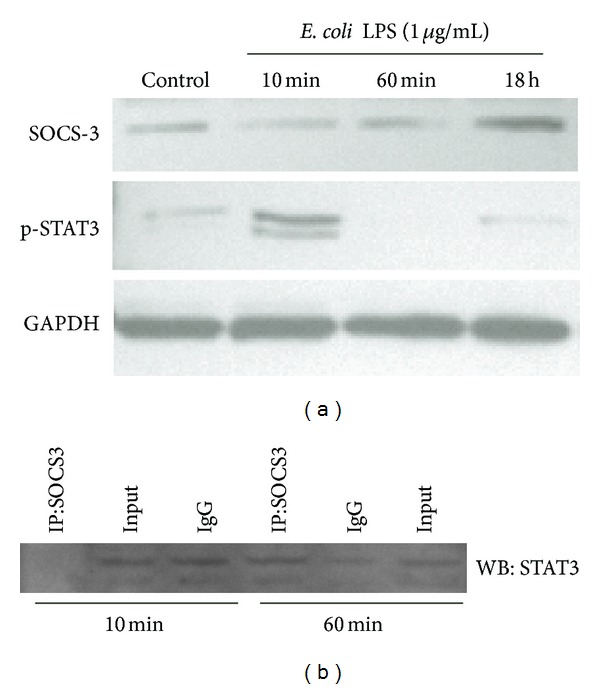
LPS stimulation transiently activates STAT3 in raw 264.7 macrophages. Expression of SOCS3 was increased only after 18 h of LPS stimulation (a). Coimmunoprecipitation indicates a dynamic direct physical interaction between SOCS3 and STAT3 in murine macrophages. Raw 264.7 cells were stimulated with LPS (or the same volume of PBS vehicle) for 10 and 60 min. SOCS3 was immunoprecipitated from 500 ug of cell lysates, and after electrophoretic separation (SDS-PAGE), STAT3 was detected by western blotting. “Input” indicates the same quantity of cell lysate loaded onto “empty” affinity columns (no primary antibody immobilized), and “IgG” represents columns in which the same quantity of irrelevant IgG from rabbit was immobilized in the columns. Image is representative of three independent experiments.

Since we used a mouse-derived cell line of macrophages for this *in vitro* experiment, we initially determined that LPS stimulation in these cells resulted in transient STAT3 activation, as indicated by the increase of STAT3 phosphorylation 10 minutes after stimulation, followed by a return to basal levels after 60 minutes (Figure 5(a)). Interestingly, SOCS3 protein levels were noticeably increased only 18 h after LPS stimulation, indicating that the basal levels of SOCS3 were sufficient to attenuate the LPS-induced activation of STAT3 60 minutes after stimulation, as well as to prevent constitutive activation of STAT3 in the absence of stimulation.

Within 10 min of LPS stimulation (Figure 5(b)), there is no physical interaction of SOCS3 and STAT3, suggesting that the endogenous negative regulation is repressed, allowing the activation of STAT3 for an appropriate cell response, as shown in Figure 5(a). STAT3-SOCS3 physical interaction was noticed 60 min after LPS stimulation, which correlated with the cessation of STAT3 activation observed in Figure 5(a).

## 4. Discussion

Experimental animal models of periodontitis are widely used for a better understanding of periodontal disease pathogenesis [14] and provide important information on inflammation associated with host-microbial interactions. Previously, our group analyzed the expression of SOCS1 and SOCS3 in ligature-induced periodontitis in rats [15]. At the protein level, the expression levels of SOCS3 accompanied the disease progression and severity of inflammation. The LPS injection model has been shown to induce inflammation in the periodontal tissues by activating innate and adaptive immune responses, which modulate the expression of various inflammatory mediators [16, 17]. Endogenous mechanisms regulating cytokine expression and biological activity are crucial to the tight regulation of the expression of inflammatory cytokines in the immune response. SOCS are a family of cytoplasmic, inducible proteins that play a role in the endogenous regulation of cytokine expression and activity. In this study we show that SOCS3 expression is increased both at the mRNA and protein levels in inflamed tissues, indicating a potential role of this gene in the pathogenesis and progression of periodontal disease. It is documented that SOCS mRNA is expressed at low levels in healthy periodontal tissues [11]. Our results from the control group are in accordance with these findings, as SOCS3 expression was lower than in the experimental group and did not vary significantly during the 30-day experimental period. In the LPS-injected gingival tissues, the positive correlation between inflammation and increased levels of SOCS3 mRNA and protein is consistent with the literature showing that the expression of SOCS is induced by LPS and inflammatory cytokines such as IL-6, IFN-*γ*, and TNF-*α*, [7, 18, 19] which are produced in response to intense antigenic challenge in periodontal disease [20–22]. It is tempting to speculate that the slight decrease on the expression of proinflammatory cytokines at 30 days, in spite of sustained challenge with LPS, was in part due to the regulatory actions of increased SOCS3; however, the possible contributions of SOCS3 in the attenuation of inflammation will be assessed in subsequent studies using approaches of gain and loss of function of SOCS3.

Interestingly, our results show that the kinetics of SOCS3 expression paralleled the severity of inflammation and bone resorption, indicating a strong association of the inflammatory status and the expression of SOCS3, suggesting the involvement of inflammatory cells or its products in the induction of SOCS expression. This possibility is further supported by the finding that SOCS mRNA and protein were not decreased in the LPS experimental model, which is characterized by sustained inflammation throughout the 30-day experimental period. This sustained inflammation is consistent with the persistent challenge to the host immune system by the injections performed 3 times per week for the duration of the experimental period. 

After inflammatory stimuli, SOCS proteins act as endogenous negative regulators of inflammation attenuating cytokine-induced signal transduction affecting primarily the JAK-STAT pathway, as part of a negative feedback loop to suppress the downstream effects of cytokines inhibiting the response to subsequent stimuli [5]. In our model, SOCS3 protein expression level was increased in the same periods as STAT3 total protein and its active phosphorylated form. These data suggest that increased expression of SOCS3 may represent a mechanism of negative regulation in response to activity of STAT3 and may be an important mechanism in regulating expression of genes associated with degradation of connective tissue and bone resorption in periodontal disease. 

The “specificity” of SOCS3 attenuating STAT3 has been shown indirectly by studies reporting higher and prolonged STAT3 activation *in vivo* conditional knockout animals with deletion of SOCS3 in macrophages [23], as well as in murine macrophages *in vitro* upon IL-6 stimulation [24]. Notably, this is the first study to demonstrate the physical interaction between SOCS3 and its “primary target” STAT3. We observed an inverse correlation between the physical interaction of SOCS3 and STAT3 and the activation status of STAT3 in LPS-stimulated macrophages. Relaxation of STAT3-SOCS3 physical interaction allows the activation of STAT3 upon LPS-stimulation, and the termination of signaling was correlated with the increased interaction STAT3-SOCS3, that may have prevented dimerization and nuclear translocation of STAT3. This mechanism should be confirmed by subsequent gain- and loss-of-function studies *in vivo*, but it has important implications for the modulation of inflammation using modified peptides that can emulate the physical interaction of SOCS3 with STAT3. There is also the possibility that the physical interaction with other signaling intermediates is a relevant mechanism for SOCS3-mediated indirect regulation of cell signaling pathways. Moreover, the role of SOCS3 may be complex, involving both positive and negative regulation of signaling depending on cell-type/stimulation specific conditions. Recently, in IL6-stimulated dermal fibroblasts SOCS3 has been shown to physically interact with p120 RasGAP (an endogenous inhibitor of Ras, which is an upstream activator of MAPKinases), attenuating the endogenous inhibition mediated by RasGAP and allowing for an increased activation of Ras/MAPkinases [25]. 

On the other hand, evidence suggests that, in addition to JAK-STAT, multiple signaling pathways are involved in the induction of SOCS proteins, such as ERK (extracellular signal-regulated kinase) and p38 MAPK (mitogen-activated protein kinase p38) [26, 27]. It is interesting to note that the intermittent stimulation with LPS in our study resulted in a sustained activation of p38 MAPK, which is also consistent with the increased levels of inflammatory cytokine expression and inflammation. The increased phosphorylation of p38 MAPK was also correlated with an increase in SOCS3 expression. In fact, data from the literature show that activation of p38 MAPK is required for stabilization of SOCS3 mRNA and, consequently, increased SOCS3 protein expression [28]. Thus, the same signaling pathways that are negatively regulated by SOCS3 can also be involved in the induction of this very gene, suggesting the plasticity of the intracellular signaling network.

## 5. Conclusion

JAK/STAT pathway has a fundamental role in the onset and progression of various inflammatory diseases. This pathway can affect the expression of various genes with proinflammatory activity, and SOCS proteins are important endogenous negative regulators of this pathway. In the current study, we have demonstrated for the first time the dynamics of SOCS3 expression during experimental LPS-induced periodontal disease and its association with the severity of inflammation and the level of proinflammatory cytokine expression, as well as with the activation status of STAT3 and p38 MAPK signaling pathways. We have also shown, for the first time, the dynamic direct physical interaction of SOCS3 and STAT3 in LPS-stimulated macrophages, indicating this as a mechanism involved in the endogenous regulation of STAT3 activation. This information enhances the understanding of the role of SOCS3 on inflammatory conditions associated with host-microbial interactions and also provides novel information on the mechanism of SOCS3-mediated regulation of STAT3 activation. Knowledge derived from this and subsequent studies may be useful in providing diagnostic, prognostic, and even therapeutic insights for other chronic inflammatory conditions involving host-microbial interactions or even aseptic inflammation (e.g., rheumatoid arthritis).

## Figures and Tables

**Table 1:** Inventoried TaqMan Primers and probe (TaqMan Gene Expression Assays, Applied Biosystems).

Target gene	Assay ID	Acession no.	Amplicon (bp)
GAPDH	Rn99999916_s1	NM_017008.3	87
IL1-*β*	Rn00580432_m1	NM_031512.2	74
TNF-*α*	Rn01525859_g1	NM_012675.2	92
IL-6	Rn99999011_m1	NM_012589.1	90
IL-10	Rn00563409_m1	NM_012854.2	70
SOCS3	Rn00585674_s1	NM_053565.1	73
